# Is There Any Relationship between *Trichomonas vaginalis* Infection and Male Urethritis Risk? A Systematic Review and Meta-Analysis

**DOI:** 10.1155/2022/8359859

**Published:** 2022-09-06

**Authors:** Hajar Ziaei Hezarjaribi, Reza Saberi, Mahdi Fakhar, Najmeh Sadeghian

**Affiliations:** ^1^Iranian National Registry Center for Lophomoniasis (INRCL) and Toxoplasmosis (INRCT), School of Medicine, Imam Khomeini Hospital, Mazandaran University of Medical Sciences, Sari, Iran; ^2^Student Research Committee, Mazandaran University of Medical Sciences, Sari, Iran

## Abstract

**Background:**

Male urethritis is one of the most common genital tract syndromes. Though the number of patients with urethritis is increasing worldwide, the cause of many cases of non-gonococcal urethritis (NGU) is still unknown.

**Objectives:**

This study aimed to delineate the association between *Trichomonas vaginalis* (*T. vaginalis*) infection and male urethritis.

**Methods:**

The literature was searched in PubMed, Scopus, and Web of Science databases using the search terms “urethritis,” “*Trichomonas vaginalis*,” “trichomoniasis,” and “male urethritis” up to February 2020. Overall risk difference(RD) was applied to assess the relationship between *T. vaginalis* infection and male urethritis.

**Results:**

In total, seven articles were included in this systematic review and meta-analysis study. Our meta-analysis involved the review of case-control studies, including 2,242 urethritis cases and 929 individuals as controls. Among subjects examined for trichomoniasis, in the case group, 211 males were infected, and in the control group, 32 individuals were infected. The overall risk difference (RD) was 0.06, and the total reported *p* value was 0.00001. Although the result of our meta-analysis was not significant, it was shown that the risk of urethritis is 0.06 more in trichomoniasis patients than in the non-exposed group.

**Conclusion:**

Findings from the included papers showed that trichomoniasis is not a risk factor for male urethritis. Although trichomoniasis alone is not the main cause of urethritis, it can be considered one of the risk factors in male urethritis. Therefore, in the future, it is necessary to perform further studies to clarify the detailed association between *T. vaginalis* infection and urethritis risk in male patients.

## 1. Introduction

Male urethritis is one of the most common genital tract syndromes, which involves urethral inflammation [1]. It is defined as gonococcal urethritis (GU) when *Neisseria gonorrhoeae* is detected or non-gonococcal urethritis (NGU) when it is not [1, 2]. Urethritis is often asymptomatic, but symptoms of urethritis in men usually include urethral discharge, penile itching or tingling, and dysuria. It often develops due to infectious pathogens like *Chlamydia trachomatis*. *Trichomonas vaginalis* (*T. vaginalis*) and *Mycoplasma genitalium* are commonly recognized pathogens confirmed to exhibit pathogenicity to the urethra in men [3–5].

*T. vaginalis* is a flagellated protozoan parasite and is the most common non-viral infection, which can be transmitted sexually. Accordingly, almost 50% of all sexually transmitted infections (STIs) caused by this parasite are curable [6]. As mentioned, *T. vaginalis* can also cause non-gonococcal urethritis, which can lead to diseases like epididymitis, prostatitis, and chronic prostatitis in men [6, 7]. In acute or mildly symptomatic infections in male patients with trichomoniasis, the most common symptoms are clear or purulent discharge and dysuria, often associated with itching and burning sensation within the urethra. On the other hand, *T. vaginalis* also plays a crucial role as an amplification factor of HIV in developing countries [8, 9].

Although the number of patients with urethritis is increasing worldwide, the cause of many cases of NGU is still unknown [10]. On the other hand, the etiology of the disease could change its clinical features and risk factors, and based on that, we could find the best prevention and treatment protocols [11]. In addition, the results of the available original articles are contradictory. Therefore, the factors involved in urethritis and their impacts must be fully identified. To this aim, we designed a study in order to delineate the association between *T. vaginalis* infection and male urethritis.

## 2. Methods

### 2.1. Design and Search Strategy

**Figure 1:**
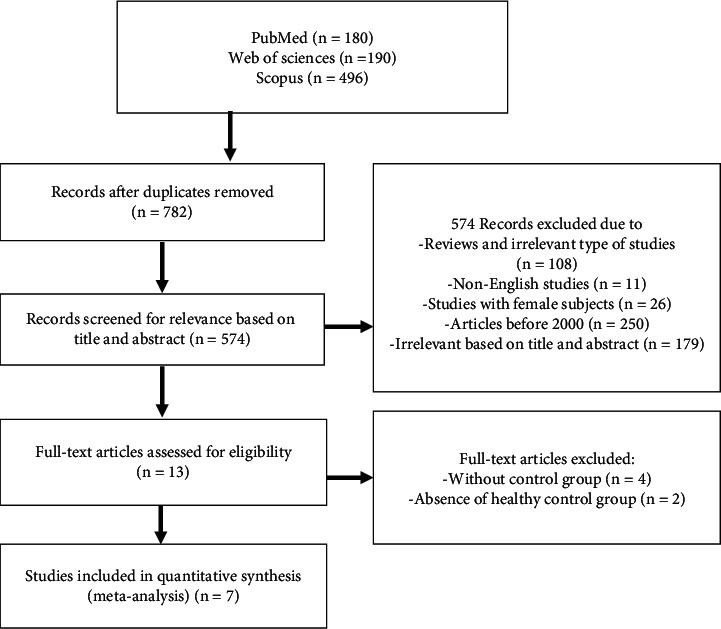
PRISMA flowchart to describe the summaries of included/excluded studies.

Preferred Reporting Items for Systematic Reviews and Meta-Analyses (PRISMA) was used for performing this study (Figure 1). The relevant case-control studies investigating the association between trichomoniasis and male urethritis up to February 2020 were systematically searched in international databases, including PubMed, Scopus, and Web of Science. The search was conducted by using the following keywords in combination or alone: “urethritis,” “*Trichomonas vaginalis*,” “trichomoniasis,” and “male urethritis.”

### 2.2. Inclusion and Exclusion Criteria

We included articles based on the following criteria: (1) case-control articles investigating the relationship between the presence of *T. vaginalis* as exposure and urethritis as a disease, (2) control and case groups were available separately, (3) original articles with full text available, and (4) presence of *T. vaginalis* detected based on validated laboratory methods like PCR. On the other hand, the exclusion criteria entailed the following: (1) included article types of reviews, books, book chapters, and letters, (2) studies not representative for our target population, (3) studies with other reasons for urethritis except of *T. vaginalis*, (4) published articles before January 2000, and (5) studies in a language other than English.

### 2.3. Data Extraction

To extract variable data, we designed a spreadsheet including the name of the first author, year of publication, study region, the age of the participants, and number of positive and negative individuals in case and control groups. The quality of the articles was assessed independently using the Newcastle-Ottawa Scale (NOS). The quality scale was within the range of 0–9 points with a score of ≤3 representing a low-quality study.

### 2.4. Statistical Analysis

**Figure 2:**
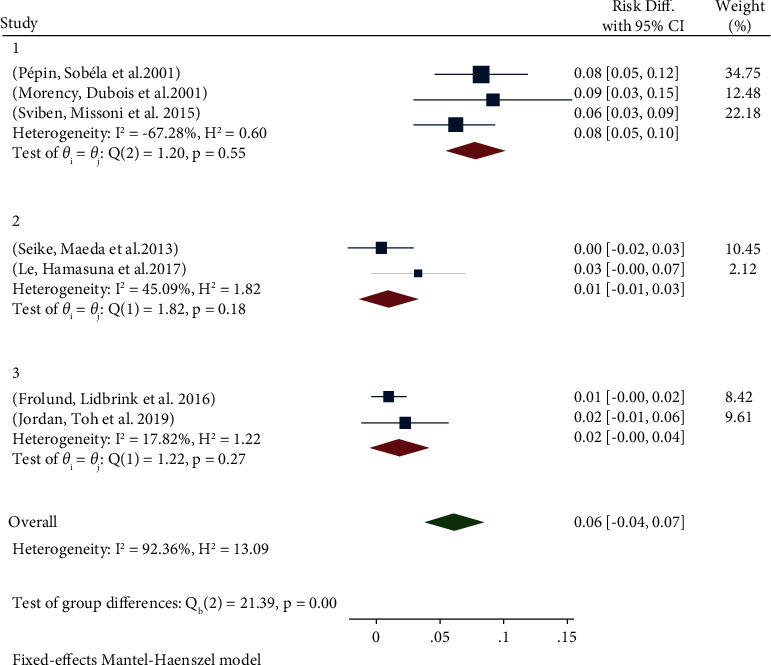
Forest plot of prevalence rate of *T. vaginalis* in male urethritis patients.

**Figure 3:**
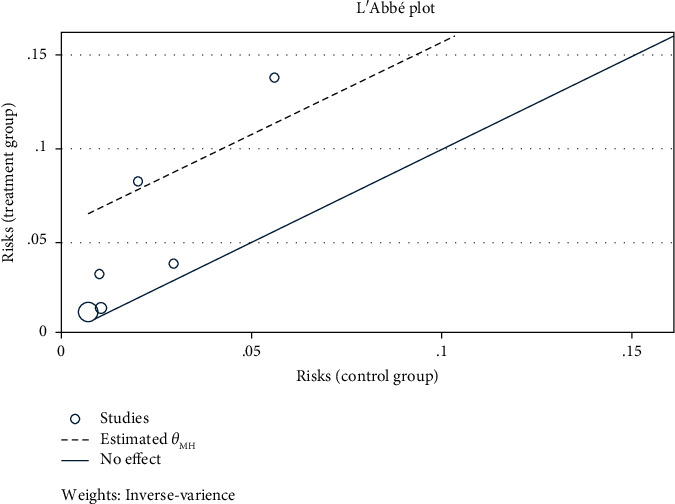
L'Abbé plot event rate in the case group against the control group.

After extract data due to the zero number of infected subjects in the control group, we calculated risk difference (RD) instead of odds ratio (OR) and their respective 95% CIs for included studies to estimate the risk of *T. vaginalis* (Figure 2). Based on the result of the chi test and heterogeneity in cases, we decided to use the Mantel–Haenszel fixed effects method in subgroup analysis. Based on the type of cases and for assessing the heterogeneity of effect estimates, we used the L'Abbé plot (Figure 3). All steps were performed using STATA software version 16.0 (Stata Corp., College Station, TX).

## 3. Result

In this systematic review, 866 studies were initially identified from the databases to organize the resources used in the research process. Initially, 82 articles were removed due to duplicate studies. In the next step, the titles and abstracts of full texts were independently reviewed and preliminary screening excluded 574 studies. Finally, 13 articles were selected for the accurate evaluation of full texts, out of which 7 articles met our criteria and were suitable for quantitative synthesis (see details in Figure 1 and Table 1).

A total of 3,171 men who had a history of urethritis were investigated for trichomoniasis, out of which 243 males were infected, distributed among 211 individuals in the case group and 32 individuals in the control group (details in Table 2). To date, the investigation of *T. vaginalis* infection and male urethritis has been studied in 5 countries. Those studies belonged to Canada (2 studies), Japan (2 studies), USA, Denmark, and Croatia (one study). All seven included studies used the PCR method for the detection of *T. vaginalis* (details in Table 2). According to the results, the mean NOS scale was 7.

The results of the forest plot demonstrated that the overall risk difference (RD) was 0.06 (95% CI, 0.04 to 0.07) and the total reported *p* value was 0.00001 (Figure 2). In the first subgroup of individuals with urethritis symptoms like urethral discharge, the RD was 0.08, which is higher than other subgroups and the overall risk difference. In the second subgroup of individuals confirmed as urethritis in cases and the third subgroup of individuals confirmed as NGU in cases, RD was about zero and presented no relationship between *T. vaginalis* infection and urethritis.

## 4. Discussion

Urethritis is common due to infection by bacteria and viruses. The infection may be from a sexually transmitted infection. Bacteria and viruses that commonly cause urethritis include *Gonococcus*, *Chlamydia trachomatis,* and herpes simplex virus (HSV-1 and HSV-2). On the other hand, *T. vaginalis* is another cause of urethritis. It is a protozoan parasite that is one of the most common non-viral STIs worldwide.

The results of the available original articles separately are contradictory, which is probably a result of the nature of *T. vaginalis*. For example, the prevalence of *T. vaginalis* depends on different factors; sexual behavior, educational level, race, socio-cultural status, and economic situation are some of the main reasons [19, 20]. Therefore, this systematic review and meta-analysis investigated the relationship between *T. vaginalis* and male urethritis. After a search in the international database, eventually seven articles were found qualified for our meta-analysis. Some researchers stated a weak association between trichomoniasis and urethritis [12, 14] while others declared *T. vaginalis* as one of the principal etiological agents of urethral discharge [13, 15]. These differences refer to the various conditions and populations of each study, which differ in many aspects of demographic features. Other reasons may be different pathogens that the person is involved in, whether identified or not.

Based on the results of the meta-analysis, the overall risk difference (RD) was 0.06 (95% CI, 0.04 to 0.07). Although the result of our meta-analysis was not significant, it was shown that the risk of urethritis is 0.06 more in trichomoniasis patients than in the non-exposed group. A recent meta-analysis study concluded the relevance between trichomoniasis and cervical cancer [21]. The findings from this study showed that individuals infected with *T. vaginalis* have a higher risk of cervical cancer in women, especially if they are co-infected with the human papillomavirus [21]. In addition, the authors stated that there is significant regional and racial variation in the correlation between trichomoniasis and the risk of cervical cancer [21].

Another systematic review and meta-analysis study surveyed the association between *T. vaginalis* infection and the risk of prostate cancer development. This research also mentioned that *T. vaginalis* could be a risk factor for prostatic cancer, and the odds of trichomoniasis in prostatic cancer patients were 1.17 times those of control individuals with a 95% CI: 1.01 to 1.36. However, it was not statistically significant [22]. In accordance with the aforementioned studies, previous studies indicated that *T. vaginalis* could cause genitourinary syndrome in men in clinical practice [23, 24].

Several limitations and data gaps restricted our study and biased our results. First, the effect of *T. vaginalis* can be obtained and detected more coherently in longitudinal cohort studies while our analysis focused on case-control papers. Second, only a low number of articles were included. Third, there are various confounding factors and parameters that we did not consider in the final analysis, like parasite symbionts, coinfections, history of previous parasitic diseases, and drug-resistant isolates.

## 5. Conclusion

In conclusion, the present study showed that although trichomoniasis alone is not the main cause of urethritis, it can be considered as a risk factor for this disease. Therefore, further studies should be conducted to assess the potential interaction between *T. vaginalis* infection and urethritis risk. In addition, future studies may confirm that *T. vaginalis* is a possible risk factor.

## Figures and Tables

**Table 1:** Baseline characteristics of the included studies in the systematic review and meta-analysis of the relationship between *T. vaginalis* infection and male urethritis patients.

Ref.	Year	Country	No. of cases	No. of controls	Age average (cases)	Age average (controls)	Groups	Diagnostic test	RD
[12]	2001	Canada	659	339	25	23	Urethral discharge	PCR	0.091
[13]	2001	Canada	410	100	27	29	Urethral discharge	PCR	0082
[14]	2013	Japan	215	98	24	27	Urethritis	PCR	0.004
[15]	2015	Croatia	500	200	nm	Nm	Urethritis symptoms	PCR	0.062
[16]	2016	Denmark	211	73	28	28	NGU	PCR	0.009
[17]	2017	Japan	92	16	34	33	Urethritis	PCR	0.033
[18]	2019	USA	155	103	28	28	NGU	PCR	0.023

**Table 2:** The number of cases and control groups with trichomoniasis.

Study (ref)	Number of cases with trichomoniasis		Number of controls with trichomoniasis	
	Positive	Negative	Positive	Negative
Morency et al. [12]	66	344	7	93
Pépin et al. [13]	91	568	19	320
Seike et al. [14]	3	212	1	97
Sviben et al. [15]	41	459	4	196
Frolund et al. [16]	2	209	0	73
Le et al. [17]	3		0	16
Jordan et al. [18]	5	150	1	102
Total	211	1942	32	897

## Data Availability

The data are available from the corresponding author upon request.
